# Characterization of Ethyl Butyrate–Induced Cough Before and After Breath Control Techniques in Healthy Adults

**DOI:** 10.1044/2022_AJSLP-22-00233

**Published:** 2023-01-12

**Authors:** Carolyn K. Novaleski, Karen Wheeler Hegland, Mikala M. Aleksandruk, Pamela H. Dalton, Joel D. Mainland

**Affiliations:** a Monell Chemical Senses Center, Philadelphia, PA; b Department of Speech, Language, and Hearing Sciences, University of Florida, Gainesville; c Health Services, College of General Studies, University of Pittsburgh, PA; d Department of Neuroscience, University of Pennsylvania, Philadelphia

## Abstract

**Purpose:**

Methods for cough elicitation frequently involve aerosolized tussive agents. Here, we sought to determine whether healthy individuals demonstrate a quantifiable cough response after inhaling a volatile ester and if breath control techniques modify this chemically induced cough response.

**Method:**

Sixty adult male and female participants inhaled prepared liquid dilutions of ethyl butyrate dissolved in paraffin oil at 20%, 40%, and 60% v/v concentrations in triplicate, with presentation order randomized. We delivered stimuli through a face mask connected to an olfactometer and respiratory pneumotachograph. Participants rated sensations of their urge to cough and pleasantness of the odor while cough airflow was measured. Following baseline testing, participants were randomized to implement pursed-lip breathing or slow-paced breathing after inhaling ethyl butyrate to determine the effects of breath control on cough measures.

**Results:**

Inhaled ethyl butyrate elicited cough in 70% of participants. Higher concentrations of ethyl butyrate resulted in significantly greater sensation of the urge to cough, *F*(2, 80) = 10.72, *p* < .001, and significantly more generated coughs, *F*(2, 63) = 13.14, *p* < .001. Compared to baseline, participants rated significantly decreased urge to cough during breath control techniques, *F*(1, 40) = 11.01, *p* = .0019. No significant changes were observed in the number of generated coughs between baseline and breath control techniques, *F*(1, 31) = 7.23, *p* = .01.

**Conclusions:**

Airborne ethyl butyrate is a tussigenic agent in humans. Our findings provide opportunities for future research directions in normal and disordered cough responses to volatile compounds.

Cough involves a well-coordinated sequence of respiratory maneuvers necessary for adequate airway protection. Specifically, the respiratory act of coughing includes initial inspiration, laryngeal compression to permit the buildup of air pressure, and expulsion to forcefully eject material from the airway (Fontana & Lavorini, 2006). Dystussia refers to pathological cough and can manifest as hypotussia and hypertussia (Spinou, 2018). In hypotussia, cough can be markedly reduced or absent and is typically observed in patients with various neurological injuries and neurodegenerative diseases (Hegland & Sapienza, 2013). Because hypotussic cough disorders are characterized by diminished airway sensations and impaired cough mechanics (Pitts et al., 2008; Troche et al., 2014), hypotussia has detrimental health implications primarily due to airway protection deficits that put patients at risk of uncompensated or undercompensated aspiration (Pitts et al., 2010; Troche et al., 2016). In contrast, hypertussia refers to conditions where too much coughing is produced, including cough hypersensitivity syndrome (CHS; Chung et al., 2022). CHS is characterized by excessive cough in response to innocuous chemical, thermal, and mechanical stimuli (Morice et al., 2014; Sundar et al., 2021). CHS poses a significant burden to patients, as its physical sequelae include rib fracture, hernia, syncope, and stress urinary incontinence (Dicpinigaitis, 2021; Schattner, 2020). Moreover, CHS has serious psychosocial implications and is associated with anxiety and depression symptoms (Dicpinigaitis et al., 2006; French et al., 2017).

Traditional research methods of eliciting cough include inhalation challenges via aerosol delivery of tussigenic agents, primarily capsaicin and citric acid (Mai et al., 2020; Morice et al., 2007; Pullerits et al., 2014; Wallace et al., 2019). Advantages of using capsaicin in cough inhalation challenges are safety and reliability (Dicpinigaitis, 2007), as well as a known receptor mechanism. Specifically, capsaicin activates the polymodal nociceptor, transient receptor potential vanilloid Type 1 ion channel (Nagy et al., 2014). Likewise, citric acid and other acid agents provoke cough with high reproducibility (Mai et al., 2020). Additional nonacid tussive stimuli include mannitol, allyl isothiocyanate, cinnamaldehyde, aerosolized adenosine 5′-triphosphate, hypertonic saline, and ultrasonically nebulized distilled water (Birrell et al., 2009; Hegland, Troche, et al., 2016; Koskela et al., 2020; Mai et al., 2020). The benefits of cough inhalation challenges using tussigenic agents are multifold, as these methods allow researchers to quantify respiratory and cough sensations (Dicpinigaitis et al., 2012) in addition to airflow measurements during cough production (Feinstein et al., 2017). Research using tussive agents has resulted in critical discoveries about cough in humans, specifically elucidating the neuronal pathways of cough (Mazzone et al., 2011), validating cough measures as predictors of dysphagia (Troche et al., 2016), and revealing impaired cough suppression in CHS (Cho et al., 2019).

While the majority of available human cough methodologies rely almost exclusively on aerosolized, nebulized, and distilled deliveries of tussigenic agents, there is growing interest in the use of alternative stimuli for more clinically feasible airway provocation challenge tests (Marcinow et al., 2015; Vertigan & Gibson, 2016). Our group previously synthesized relevant topics from the chemosensory sciences in the context of abnormal laryngeal–respiratory function associated with inhaled airborne chemical stimuli (Novaleski et al., 2021). We uncovered substantial inconsistencies in provocation challenge test stimuli, concentrations, and delivery methods, leading to difficulty with diagnostic interpretation. Although it has been postulated that quantifying chemical stimuli is problematic for inhalation challenges (Vertigan & Gibson, 2016), research methods in the chemosensory sciences actually provide relevant directions to assist with standardizing chemical stimuli for clinical assessment procedures (Novaleski et al., 2021). Moreover, tussigenic agents such as capsaicin have therapeutic applications across the continuum of disordered cough, specifically with nonpharmacological interventions in the form of behavioral speech therapy (Borders et al., 2022; Hegland, Davenport, et al., 2016; Slovarp et al., 2022). The ability to easily use nonaerosolized stimuli outside clinical settings may enhance therapeutic benefits and patient adherence, as demonstrated with exposures to odorant stimuli during olfactory training for postviral olfactory dysfunction (Altundag et al., 2015; Saatci et al., 2020; Yuan et al., 2021). Finally, methodologies using additional chemical stimuli may elucidate important factors that mediate cough in health and disease. For instance, inhaling ethanol leads to significantly more coughs during subsequent capsaicin inhalation among individuals with airway sensory hyperreactivity compared to controls (Millqvist et al., 2008), while menthol and sucrose alter cough sensitivity in healthy individuals (Wise et al., 2012).

Ethyl butyrate (EB) is a volatile ester that is found naturally in foods such as kiwi fruit and blue cheese (Bartley & Schwede, 1989; Day & Anderson, 1965). In addition to natural ingestion exposures, EB is manufactured as a flavor ingredient in consumer products such as chewing gum and candy (Burdock, 2010). At low concentrations, the odor and taste perceptions of EB are characterized as sweet or fruity (Burdock, 2010). However, inhalation of EB at higher concentrations produces irritating sensations in the upper airways that can lead to cough based on our group's prior anecdotes working with the compound. Therefore, the primary purpose of this study was to determine whether airborne delivery of EB elicits cough in healthy individuals by measuring psychophysical properties and cough airflow parameters.

Breath control techniques are a major component of behavioral speech therapy for CHS (Carroll, 2019; Vertigan & Gibson, 2016), either with or without irritant exposures (Hodges, 2012). The purpose of instructing patients in breath control maneuvers is to avoid coughing or interrupt the cough while it occurs (Vertigan et al., 2019). This goal is achieved by changing breathing patterns to permit air to flow openly throughout the respiratory tract and reduce laryngeal constriction (Murry & Milstein, 2016; Vertigan et al., 2019). Examples of common breath control techniques include pursed-lip breathing (PLB) and relaxed throat breathing (Slinger et al., 2019; Vertigan et al., 2008). Although speech therapy results in favorable clinical outcomes for CHS, it remains unknown which aspect of treatment is driving this change (e.g., breath control, laryngeal hygiene education, and psychoeducational counseling; Wamkpah et al., 2022). Research using tussigenic agents is one approach to understand how normal and pathological cough can be modified using various cognitive and behavioral techniques (Brandimore et al., 2017; Janssens et al., 2014; Perry & Troche, 2019). To further investigate the feasibility of our cough paradigm, a secondary purpose of this study was to examine the effects of breath control on cough measures after EB exposure.

## Method

### Participants

The University of Pennsylvania Institutional Review Board approved the study procedures (#829504). Enrolled participants were 60 adults (24 men, 36 women) between 18 and 35 years of age. Participants self-reported no history of respiratory, cardiovascular, neurological, cough, swallowing, voice, or olfactory problems; chemical sensitivities; occupational chemical exposures; smoking; gastroesophageal or laryngopharyngeal reflux; and surgery or trauma to the head, neck, or larynx. Female participants reported that they were not pregnant or lactating. After providing verbal and written informed consent, participants completed the following questionnaires: Voice Handicap Index (Jacobson et al., 1997), Newcastle Laryngeal Hypersensitivity Questionnaire (Vertigan et al., 2014), and Reflux Symptom Index (Belafsky et al., 2002). Upon study completion, they received financial compensation for their participation.

### Procedure

**Figure 1.**
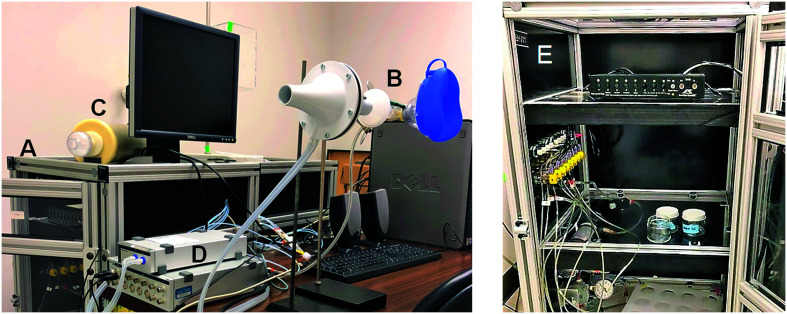
Experimental setup. (A) Outside view of the eight-channel, computer-controlled dynamic dilution olfactometer that delivers chemical stimuli. (B) Spirometer differential pressure transducer connected to a pneumotach flow head and face mask. (C) Calibration syringe. (D) Data acquisition hardware unit that records and digitizes airflow signals. (E) Inside view of the olfactometer.

Liquid solutions of EB (Sigma-Aldrich, CAS No. 105-54-4) were diluted in paraffin oil (Sigma-Aldrich, CAS No. 8012-95-1) at 20%, 40%, and 60% v/v concentrations, with a total volume of 10 mL prepared in 1-oz straight-sided glass jars (Uline). EB was delivered using a computer-controlled, eight-channel olfactometer (see Figures 1A and 1E; Lundström et al., 2010). Pressurized air was dried using Drierite (W. A. Hammond Drierite) and then passed over active carbon to remove impurities and residual chemical odors. Dry-filtered air entered the olfactometer via polyurethane tubing and was routed to independent channels connected to glass jars containing EB-paraffin oil liquid solutions, at which point the air became mixed with the stimuli. Headspace vapor, or the equilibrated airborne concentration of the chemical above the liquid solution, was delivered at a flow rate of 3 L/min through the glass jars connected to fluoropolymer tubing. The stimuli were delivered to participants through a silicone face mask for 3 s per trial. Instructions and stimuli were presented using custom software written using E-Prime (Version 2.0, Psychology Software Tools).

Prior to the experiment, participants were initially exposed to EB in ascending order of low to high concentrations until an audible cough was produced. This task familiarized participants with the stimuli, ensured they were comfortable with the task, and determined if they demonstrated a cough response to EB. Next, all participants received standardized verbal instructions from the research team about the experiment. Participants were instructed that they would inhale a stimulus through the silicone face mask and were explicitly told to “cough directly into the face mask,” which was connected to a pneumotachograph (see Figure 1B). Participants received additional verbal instruction to “breathe through your nose,” “cough if you need to cough,” and “do not hold back a cough” to discourage participants from suppressing cough. Participants were also informed that they would take breaks without wearing the face mask between trials.

Baseline experimental testing involved the presentation of three EB concentrations in triplicate in a randomized and blinded manner, resulting in a total of nine trials. During trial presentations of the stimuli, participants attended to a computer monitor while wearing the silicone face mask that provided visual and auditory prompts to inhale on a countdown to synchronize the temporal delivery of the stimulus. An auditory tone was presented for 3 s while the chemical stimulus was delivered to participants, at which time participants inhaled for 3 s. No prompts to exhale were provided. Following the stimulus delivery, no subsequent computer prompts were provided about the duration or rate of inhalation or exhalation. Participants continued to breathe through the face mask while making sensory ratings. To reduce the effects of sensory adaptation between trial presentations, participants breathed room air without the face mask for a minimum of 60-s intervals as previously described (Dicpinigaitis, 2003; Fullerton et al., 2021). No specified number of breath cycles was recorded prior to stimulus delivery.

To further investigate our cough paradigm, we tested whether the implementation of breath control influenced cough. Following a 10-min break outside the testing room to reduce the effects of sensory adaptation to EB, participants were randomized to one of two breath control conditions: PLB (*n* = 30) or slow-paced breathing (SPB, *n* = 30). Participants repeated the aforementioned cough inhalation challenge with nine trials of randomized EB concentrations presented in triplicate. Participants in both conditions received the same standardized verbal instructions during baseline testing (i.e., “cough directly into the face mask,” “breathe through your nose,” “cough if you need to cough,” and “do not hold back a cough”). The only additional instruction provided was the manner of breathing. Participants in the PLB condition were instructed to inhale by sniffing through the nose and exhale slowly through pursed lips, the rationale being that the technique reduces narrowing of the airway on expiration (Dechman & Wilson, 2004). In the SPB condition, participants were instructed to slowly inhale and exhale through the nose (Zaccaro et al., 2018). This condition was selected to serve as a control to determine whether directed attention and volitional control of breathing changed cough outcomes. The researchers monitored respiratory airflow, and if needed, participants received verbal reminders to modify their breathing.

### Outcome Measures

Psychophysical measurements were collected using E-Prime. Following each trial, participants rated the intensity of the urge to cough (UTC; Dicpinigaitis et al., 2012) using the general Labeled Magnitude Scale (gLMS; Green et al., 1996), a logarithmic rating scale with semantic labels to assess suprathreshold sensory responses. The bottom anchor was “no sensation,” and the top anchor was “strongest imaginable sensation.” Participants also rated the hedonic quality of odor pleasantness (Rouby et al., 2009). The bottom anchor was “unpleasant,” the top anchor was “pleasant,” and the middle of the scale was “neutral.”

For cough mechanics, airflow was measured using a 1,000 L/min pneumotach flow head connected to a spirometer differential pressure transducer (ADInstruments; see Figure 1B) following calibration using a 3-L calibration syringe (Hans Rudolph; see Figure 1C). Airflow signals were recorded and digitized at a sampling rate of 2000 Hz using PowerLab 8/35 data acquisition hardware unit (see Figure 1D) in conjunction with LabChart 8 software (ADInstruments). Cumulative cough frequency was calculated as the sum of all cough epochs, which also included cough reaccelerations from one inspiration (Troche et al., 2016) across triplicate trial presentations. Additional metrics were analyzed from the first cough epoch per trial, including cough latency as the duration between EB delivery and cough occurrence. Compression-phase duration was the time that airflow approximated zero preceding the expiratory phase. Peak expiratory flow rate (PEFR) was the maximum airflow during the first burst of expired air, PEFR rise time was calculated as the duration between the end of the compression phase to maximum expired airflow, and cough volume acceleration was obtained by dividing PEFR by PEFR rise time (Hegland & Sapienza, 2013).

### Statistical Analyses

Statistical analyses were performed using RStudio (Version 2022.2.1.461). Figures were generated using the data visualization package ggplot2 (version 3.3.5). For main effects, a Bonferroni-corrected *p* value was set to .002. A series of four independent *t* tests was computed to establish if there were baseline participant differences between breath control conditions. To investigate the reliability across trial presentations for UTC and odor pleasantness and to determine if there was a relationship between UTC and cumulative cough frequency, three Pearson product–moment correlation coefficients were computed. A series of three separate three-way, 3 × 2 × 2 mixed analyses of variance (ANOVAs) was performed to determine whether there were significant main effects of concentration (three levels of 20%, 40%, and 60% v/v, within variable), time (two levels of baseline testing and breath control techniques, within variable), and/or breath control condition (two levels of PLB and SPB, between variable) on UTC, odor pleasantness, and cumulative cough frequency. For additional analyses of cough airflow parameters obtained from only participants who coughed, a series of five separate two-way, 3 × 2 mixed ANOVAs was calculated to determine whether there are significant main effects of concentration (three levels, within variable) and/or sex (two levels, between variable) on cough latency, compression phase duration, PEFR, PEFR rise time, and cough volume acceleration. When significant main effects were observed, post hoc pairwise comparison tests were performed using an uncorrected *p* value of .05.

## Results

Inhaled EB was well tolerated by participants, with no adverse events reported. In total, cough was elicited among 42 out of 60 enrolled participants (70%) in response to at least one of the selected concentrations of 20%–60% v/v EB. Therefore, *N* = 42 participants were included in analyses of participant characteristics and psychophysical evaluations. The remaining 30% of participants were excluded from analysis because no coughs were generated at the tested concentrations (*n* = 9), participants desensitized to EB after coughing during the initial inhalation task in ascending order but not subsequent randomized trials (*n* = 8), and participants coughed after airflow recordings stopped (*n* = 1). No obvious differences were noted between participants who coughed to EB versus those who were excluded. For cough airflow analyses, only 34 of these 42 participants were included due to file corruption.

### Participant Characteristics

Table 1 displays group means for age and questionnaire scores. As expected, scores fell within normal limits for self-reported laryngeal function compared to previously published normative data (Arffa et al., 2012; Novaleski et al., 2011; Vertigan et al., 2014). Group equivalence was established on key variables prior to random assignment to breath control conditions, as no significant differences were observed between participants assigned to PLB versus SPB for age (*t* = −0.44, *p* = .66), Voice Handicap Index (*t* = 0.34, *p* = .74), Newcastle Laryngeal Hypersensitivity Questionnaire (*t* = −0.81, *p* = .42), and Reflux Symptom Index (*t* = −0.12, *p* = .91). Male and female participants were evenly distributed in both breath control conditions (50%).

**Table 1.** Breath control group equivalence on age and self-reported laryngeal function for participants assigned to pursed-lip breathing (PLB) and slow-paced breathing (SPB) conditions.

Characteristic	PLB (*n* = 22)	SPB (*n* = 20)	Mean difference [95% CI]	*t*	*p*
Age	27.05 ± 5.06	26.45 ± 3.50	0.60 [−3.33, 2.14]	−0.44	.66
VHI	5.64 ± 6.90	6.40 ± 7.74	−0.76 [−3.80, 5.33]	0.34	.74
NLHQ	94.00 ± 4.32	92.65 ± 6.40	1.35 [−4.73, 2.03]	−0.81	.42
RSI	2.09 ± 2.45	2.00 ± 2.66	0.09 [−1.68, 1.50]	−0.12	.91

*Note.* Data are means and standard deviations (*X* ± *SD*); *N* = 42. CI = confidence interval; VHI = Voice Handicap Index; NLHQ = Newcastle Laryngeal Hypersensitivity Questionnaire; RSI = Reflux Symptom Index.

### Psychophysical Evaluation

#### Intrasubject Reliability

Across EB trials presented in triplicate during baseline testing, significantly positive correlations were observed between Trial 1 and Trials 2 and 3 for both UTC (*r* = .45, *p* < .001) and odor pleasantness (*r* = .64, *p* < .001). These findings demonstrate acceptable intrasubject reliability for psychophysical measurements. For subsequent analyses, UTC and odor pleasantness were averaged across three trial presentations per concentration.

#### UTC

**Figure 2.**
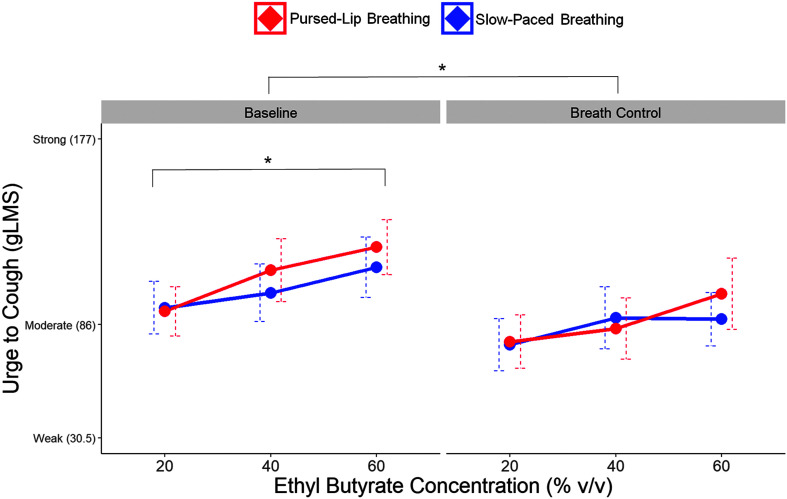
Higher concentrations of inhaled ethyl butyrate increase urge to cough, but pursed-lip breathing downregulates urge to cough to similar levels as slow-paced breathing. Dots represent mean ratings of the intensity of urge to cough with corresponding semantic labels (*y*-axis) across 20%, 40%, and 60% v/v concentrations of ethyl butyrate (*x*-axis) at baseline (left) and implementation of breath control (right). Participants in the pursed-lip breathing condition are red, and participants in the slow-paced breathing condition are blue (*N* = 42). Error bars are dashed lines that represent standard errors of the mean. *p ≤ .0019. gLMS = general Labeled Magnitude Scale.

There was a significant main effect of concentration of inhaled EB on UTC, *F*(2, 80) = 10.72, *p* < .001. Post hoc pairwise comparisons revealed that mean UTC significantly increased at 60% compared to 20% v/v concentration (107 vs. 85 gLMS ratings, *p* < .05). These ratings corresponded approximately to the semantic label of moderately intense UTC. In addition, mean UTC significantly decreased during the implementation of breath control techniques compared to baseline, *F*(1, 40) = 11.01, *p* = .0019 (86 vs. 107 gLMS ratings), but was not significantly different between PLB and SPB, *F*(1, 40) = 0.07, *p* = .79. Figure 2 shows group means for UTC across inhaled EB concentration, time, and breath control condition.

#### Hedonics

For the hedonic attribute of odor pleasantness, there were no significant main effects of concentration, *F*(2, 80) = 1.27, *p* = .29; time, *F*(1, 40) = 0.03, *p* = .87; or breath control condition, *F*(1, 40) = 0.04, *p* = .84. Across concentrations, hedonic ratings corresponded to approximately the middle of the rating scale of a neutral percept, suggesting that participants generally perceived EB as neither pleasant nor unpleasant.

### Cough Mechanics

#### Cumulative Cough Frequency

**Figure 3.**
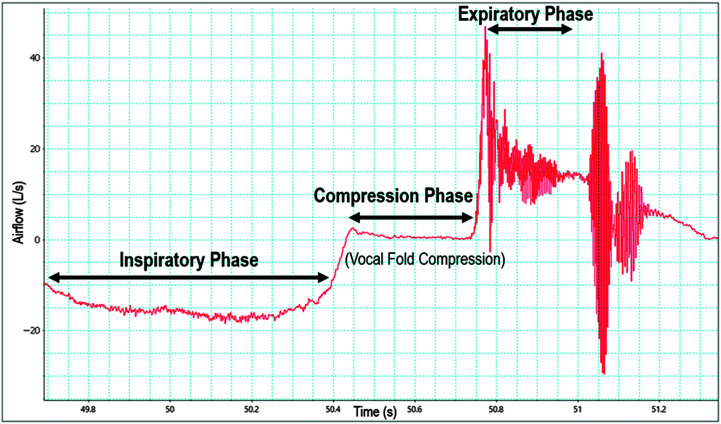
Representative cough airflow waveform. Digitized airflow waveform depicting real-time respiratory patterns of a cough. Cough airflow parameters are displayed during the inspiratory, compression, and expiratory phases.

**Figure 4.**
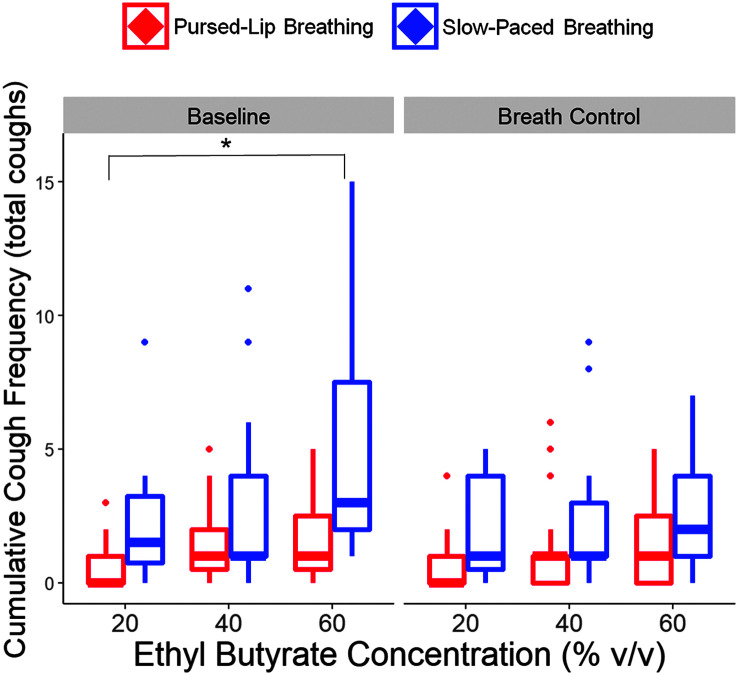
More coughs are generated with higher concentrations of inhaled ethyl butyrate. Data are represented with box plots, in which the thickest horizontal lines display median cumulative cough frequency (*y*-axis) across 20%, 40%, and 60% v/v concentrations of ethyl butyrate (*x*-axis) at baseline (left) and implementation of breath control (right). Participants in the pursed-lip breathing condition are red, and participants in the slow-paced breathing condition are blue (*N* = 34). Error bars display minimum and maximum values. *p < .001.

Figure 3 displays a representation of an airflow waveform of a generated cough in response to inhaled EB. Digitized cough airflow waveforms revealed a significant main effect of concentration of inhaled EB on cumulative cough frequency, *F*(2, 63) = 13.14, *p* < .001. Post hoc pairwise comparisons revealed that the number of coughs significantly increased at 60% compared to 20% v/v concentration (2.63 vs. 1.48 coughs, *p* = .005). Moreover, cumulative cough frequency was not significantly different following breath control techniques compared to baseline, *F*(1, 31) = 7.23, *p* = .01, nor was it significantly different between PLB and SPB, *F*(1, 31) = 3.82, *p* = .06. Figure 4 displays median cumulative cough frequency across inhaled EB concentration, time, and breath control condition. Of note, there were only weak correlations between UTC and cumulative cough frequency, *r* = .16, *p* = .02. UTC, hedonics, and cumulative cough frequency data are presented in Table 2.

**Table 2.** Psychophysical evaluation and total number of coughs produced across inhaled ethyl butyrate concentrations before and after breath control implementation for participants assigned to pursed-lip breathing (PLB) and slow-paced breathing (SPB).

Measurement	20% v/v		40% v/v		60% v/v	
	PLB	SPB	PLB	SPB	PLB	SPB
Baseline						
Urge to cough (gLMS)	92.47 ± 56.85 [67.26, 117.68]	94.17 ± 57.63 [67.20, 121.14]	112.58 ± 72.20 [80.57, 144.59]	101.50 ± 62.99 [72.20, 130.98]	123.92 ± 63.04 [95.97, 151.87]	114.08 ± 66.35 [83.03, 145.13]
Hedonics (pleasantness gLMS)	8.02 ± 103.42 [−37.83, 53.87]	10.32 ± 88.16 [−30.94, 51.58]	−4.58 ± 104.07 [−50.72, 41.56]	9.25 ± 89.29 [−32.54, 51.04]	−12.33 ± 104.56 [−58.69, 34.03]	11.03 ± 89.44 [−30.83, 52.89]
Cumulative cough frequency (total coughs)	0.72 ± 0.96 [0.29, 1.15]	2.44 ± 2.87 [1.10, 3.78]	1.53 ± 1.47 [0.88, 2.18]	2.93 ± 3.35 [1.36, 4.50]	1.68 ± 1.53 [1.00, 2.36]	5.00 ± 3.85 [3.20, 6.80]
Breath control						
Urge to cough (gLMS)	77.62 ± 61.14 [50.51, 104.73]	76.05 ± 57.36 [49.20, 102.90]	84.06 ± 70.50 [52.80, 115.32]	89.27 ± 67.99 [57.45, 121.09]	101.03 ± 81.61 [64.85, 137.21]	88.60 ± 58.53 [61.21, 115.99]
Hedonics (pleasantness gLMS)	8.68 ± 94.57 [−33.25, 50.61]	1.30 ± 83.00 [−37.55, 40.15]	0.91 ± 96.87 [−42.04, 43.86]	8.50 ± 83.21 [−30.44, 47.44]	5.52 ± 102.78 [−40.05, 51.09]	0.23 ± 94.03 [−43.78, 44.24]
Cumulative cough frequency (total coughs)	0.89 ± 1.29 [0.32, 1.46]	1.93 ± 1.83 [1.07, 2.79]	1.37 ± 1.74 [0.60, 2.14]	2.40 ± 2.75 [1.11, 3.69]	1.53 ± 1.54 [0.85, 2.21]	2.60 ± 2.16 [1.59, 3.61]

*Note.* Data are means and standard deviations (*X* ± *SD*), and 95% confidence intervals are in brackets; *N* = 42. gLMS = general Labeled Magnitude Scale.

#### Cough Airflow Parameters

Further parameters of cough airflow were analyzed on trials in which coughs occurred during baseline to better understand cough aerodynamics during inhaled EB (see Table 3). If participants coughed, the first cough epoch was analyzed at each concentration. During baseline, there were no significant differences in EB concentration on cough latency, *F*(2, 39) = 1.49, *p* = .24; compression phase duration, *F*(2, 38) = 1.34, *p* = .27; PEFR, *F*(2, 38) = 0.98, *p* = .39; PEFR rise time, *F*(2, 38) = 0.86, *p* = .43; and cough volume acceleration, *F*(2, 38) = 0.85, *p* = .43. Male participants produced significantly higher cough volume acceleration compared to female participants, *F*(1, 28) = 18.26, *p* < .001 (57.14 vs. 31.24 L/s). However, no significant sex differences were observed with cough latency, *F*(1, 28) = 0.00, *p* = .96; compression phase duration, *F*(1, 28) = 0.80, *p* = .38; PEFR, *F*(1, 28) = 5.98, *p* = .02; and PEFR rise time, *F*(1, 28) = 5.09, *p* = .03. Cough airflow parameters were not analyzed for the variables of time and breath control due to missing data from participants who did not consistently cough per trial.

**Table 3.** Cough airflow parameters for males and females across inhaled ethyl butyrate concentrations.

Measurement	20% v/v		40% v/v		60% v/v	
	Males	Females	Males	Females	Males	Females
Cough latency (s)	17.77 ± 13.23	16.01 ± 12.91	18.81 ± 15.45	19.06 ± 12.34	13.96 ± 10.92	15.55 ± 9.91
	[10.72, 24.82]	[9.59, 22.43]	[10.58, 27.04]	[12.92, 25.20]	[8.14, 19.78]	[10.62, 20.48]
Compression phase duration (s)	0.35 ± 0.31	0.69 ± 0.53	1.17 ± 2.97	0.46 ± 0.26	1.94 ± 3.25	0.87 ± 0.80
	[0.18, 0.52]	[0.43, 0.95]	[−0.41, 2.75]	[0.33, 0.59]	[0.21, 3.67]	[0.47, 1.27]
Peak expiratory flow rate (L/s)	1.92 ± 0.99	1.53 ± 0.64	2.40 ± 0.76	1.59 ± 0.89	2.25 ± 0.96	1.65 ± 0.68
	[1.39, 2.45]	[1.21, 1.85]	[2.00, 2.80]	[1.15, 2.03]	[1.74, 2.8]	[1.31, 1.99]
Peak expiratory flow rate rise time (s)	0.05 ± 0.02	0.06 ± 0.02	0.04 ± 0.01	0.06 ± 0.03	0.04 ± 0.01	0.05 ± 0.02
	[0.04, 0.06]	[0.05, 0.07]	[0.03, 0.05]	[0.05, 0.07]	[0.03, 0.05]	[0.04, 0.06]
Cough volume acceleration (L/s)	43.51 ± 22.52	31.13 ± 19.05	61.88 ± 28.31	29.11 ± 16.49	58.80 ± 30.58	33.31 ± 11.15
	[31.51, 55.51]	[21.66, 40.60]	[46.79, 76.97]	[20.91, 37.31]	[42.51, 75.09]	[27.77, 38.85]

*Note.* Data are means and standard deviations (*X* ± *SD*), and 95% confidence intervals are in brackets; *N* = 34 (16 males, 18 females).

## Discussion

A wide body of literature exists on the delivery of various stimuli during cough inhalation challenges (Mai et al., 2020; Morice et al., 2007; Pullerits et al., 2014; Wallace et al., 2019). Despite valuable advancements in human cough inhalation challenges using primarily aerosolized tussigenic agents such as capsaicin and citric acid, a variety of other stimuli and delivery methods are used in clinical practice during airway provocation challenge tests (Novaleski et al., 2021). Current experimental methods using aerosolized tussive agents do not completely represent the broad spectrum of potential clinical uses of cough-inducing stimuli (Sandage et al., 2021). In particular, conducting informal assessments using airway and cough challenges is advantageous when preparation and administration are clinically practical (Vertigan & Gibson, 2016). In addition, increasingly innovative approaches with tussigenic agents are being implemented in nonpharmacological treatments for cough disorders (Borders et al., 2022; Hegland, Davenport, et al., 2016; Slovarp et al., 2022). Expanding research methods in airborne irritants and tussive stimuli may further elucidate the mediating factors and mechanisms underlying normal and pathological cough. For additional examples of the clinical implications of using chemical stimuli in laryngeal–respiratory and cough disorders, please see the study of Novaleski et al. (2021).

Here, we selected high concentrations of a volatile ester, EB, with previous applications in chemosensory and food industry research to characterize both psychophysical and physiological cough parameters via airborne inhalation using olfactometry among healthy men and women. Results demonstrated that 20%–60% v/v concentrations of EB–paraffin oil liquid dilutions elicited cough in 70% of participants in our sample. This rate of cough responsiveness appears consistent with some other tussigenic agents. Similar rates of coughing in healthy adults have been reported to inhaled citric acid (80% of participants at 32% concentration or below; Midgren et al., 1992). Although fewer healthy adults (17%) coughed at lower concentrations of inhaled capsaicin (2 and 10 μM; Midgren et al., 1992), all normal participants in another sample coughed to capsaicin across a larger range of concentrations (0.49–1000 μM; Dicpinigaitis et al., 2012). It is important to note that not all published studies indicate whether enrolled participants were initially screened to determine if they cough to tussive stimuli, which could influence the known percentage of cough responders.

Participants reported no adverse events with inhaled EB. Volatile esters have been deemed safe for use in foods (Dinu et al., 2020). The hedonic quality of EB was further characterized as neutral, or neither pleasant nor unpleasant. UTC, as measured by intensity suprathreshold scaling using the gLMS, was reasonably reliable at the tested concentrations within a single testing session. UTC increased in a dose-dependent manner, with higher intensities of UTC occurring with higher concentrations. Given that the percept of odor pleasantness remained unchanged, UTC appears to be a specific psychophysical metric associated with EB inhalation. Moreover, we quantified objective motor parameters of the cough response. Results revealed a similar dose–response with cough frequency across EB concentrations, such that more coughs were generated with higher concentrations. However, EB concentration did not change cough airflows. Beyond characterizing how inhaled EB elicits reflex cough, findings from this cough paradigm indicated that modifying respiration in the form of two separate breath control techniques after EB exposure attenuated UTC. Of note, UTC was decreased to similar levels for participants assigned to both PLB and SPB conditions.

To our knowledge, this is the first study to use dynamic olfactometry, a highly controlled system for delivering odorant stimuli in smell research (Lundström et al., 2010), to deliver a volatile compound with the intent to elicit cough. Vapor ammonia has previously been used to elicit the glottic stop, an upper airway reflex, but does not appear to induce cough in normal controls (Langton et al., 1993; Prudon et al., 2005). Our findings unlock new opportunities for expanding cough research methodologies that do not require aerosol deliveries of stimuli. There are several potential directions for future research using this cough model. From a basic research perspective, cough thresholds could be obtained for EB to determine the normal range of cough sensitivity to this compound between males and females, as well as across a range of age groups. Another useful research endeavor would be to directly compare cough responses to EB using dynamic olfactometry, as was used in this study, to low-technology delivery systems (e.g., sniff bottles; Smeets et al., 2007). More practical routes of delivering chemosensory stimuli such as EB have important implications for implementation in research and clinical settings.

Given that EB is airborne, additional methodologies from the chemosensory sciences provide further guidance in addressing important factors in human psychophysics experiments. Namely, olfactory and other forms of sensory adaptation to EB may occur (Pellegrino et al., 2017). Future work could investigate the individual variability in desensitization, as well as sensitization, to EB-induced cough. Desensitization to aerosolized capsaicin has previously been investigated as a therapeutic approach in patients with CHS (Slovarp & Bozarth, 2019; Slovarp et al., 2022). More systematic methods regarding temporal aspects of intertrial presentations and durations of exposure of tussigenic stimuli are warranted to enhance understanding of the normal sensory adaptation of cough to subsequently interpret observations in clinical populations.

In addition to further validation studies using chemosensory stimuli to elicit cough, there are clinical implications. Given that as many as 214 cough triggers were identified among patients with CHS (Sandage et al., 2021), we speculate that perhaps some patients with CHS demonstrate consistently higher UTC and greater cough frequency to multiple inhaled airborne compounds commonly used in olfactory research at lower concentrations compared to healthy individuals. Precise control over concentrations of a battery of airborne compounds may help elucidate measurable differences between individuals with and without CHS, such as better defining the nature of cough symptoms in response to reportedly innocuous stimuli (Morice et al., 2014; Sundar et al., 2021). Moreover, further research is needed to determine if such methods are useful to better distinguish which etiologies (e.g., higher cognitive vs. neurobiological mechanisms) are likely driving cough symptoms across clinical subtypes of CHS. Although the cough measure of cumulative cough frequency was statistically significant in the current investigation, the clinical significance of this measure and others remains to be fully understood until further work is pursued with patients. Finally, while the mechanism underlying cough elicited by EB remains unknown, our data support the argument that there are multiple stimuli that activate various cough receptors throughout the airway (Koskela et al., 2020).

Our study failed to find a significant difference in cumulative cough frequency between baseline breathing and breath control techniques. One potential explanation for this observation is that too few coughs were produced at baseline at the selected concentrations of inhaled EB. We hypothesize that higher EB concentrations might result in a more pronounced difference in cumulative coughs, as well as changes in cough airflow parameters. This hypothesis should be tested in future cough studies. Following breath control, the observed discrepancy in changes in the psychophysical properties of UTC, but not the output of cough frequency, suggests that these cough measurements provide different information. As such, clinicians who assess and treat cough disorders may benefit from viewing a patient's cough from both a sensory and motor perspective.

We readily acknowledge the limitations of this study. First, only healthy individuals without cough complaints were tested using inhaled EB in a controlled laboratory setting. This makes it challenging to generalize the present findings to patients with CHS. It remains unknown whether patients would temporarily experience reduced UTC following breath control and how long this effect would last. We advocate for further research among patients with cough disorders, in particular to adapt feasible and clinically meaningful methods with chemosensory stimuli for assessment and treatment. Additionally, future investigations are warranted in chemosensory-induced cough that targets components of speech therapy other than breath control, such as psychoeducational counseling. Such studies would better elucidate which specific aspects of behavioral interventions influence improvements in clinical cough outcomes.

## Conclusions

The results of our work indicate that the volatile compound EB, in addition to being found naturally in foods and serving as a flavor ingredient, is a tussigenic stimulus in humans. We measured the concentration–intensity function of the sensation of UTC using suprathreshold ratings and number of coughs produced after inhaling EB. Moreover, we demonstrated that breath control techniques after EB inhalation reduce UTC. To our knowledge, this is the first application of volatile EB delivery using dynamic olfactometry to induce cough. The methods described here offer unique opportunities for future research directions to investigate normal and disordered cough

## Data Availability Statement

The data sets generated during and/or analyzed during this study are available from the corresponding author on reasonable request.
